# Chromosome-scale genome assembly of the ‘Munstead’ cultivar of *Lavandula angustifolia*

**DOI:** 10.1186/s12863-023-01181-y

**Published:** 2023-12-13

**Authors:** John P. Hamilton, Brieanne Vaillancourt, Joshua C. Wood, Haiyan Wang, Jiming Jiang, Douglas E. Soltis, C. Robin Buell, Pamela S. Soltis

**Affiliations:** 1 Center for Applied Genetic Technologies, University of Georgia, Athens, GA USA; 2 Department of Crop and Soil Sciences, University of Georgia, Athens, GA USA; 3 Department of Plant Biology, Michigan State University, East Lansing, MI USA; 4 Department of Horticulture, Michigan State University, East Lansing, MI USA; 5 Department of Biology, University of Florida, Gainesville, FL USA; 6 Museum of Natural History, University of Florida, Gainesville, FL USA; 7 Institute of Plant Breeding, Genetics, and Genomics, University of Georgia, Athens, GA USA

**Keywords:** Terpene, Lamiaceae, Essential oil

## Abstract

**Objectives:**

*Lavandula angustifolia* (English lavender) is commercially important not only as an ornamental species but also as a major source of fragrances. To better understand the genomic basis of chemical diversity in lavender, we sequenced, assembled, and annotated the ‘Munstead’ cultivar of *L. angustifolia*.

**Data description:**

A total of 80 Gb of Oxford Nanopore Technologies reads was used to assemble the ‘Munstead’ genome using the Canu genome assembler software. Following multiple rounds of error correction and scaffolding using Hi-C data, the final chromosome-scale assembly represents 795,075,733 bp across 25 chromosomes with an N50 scaffold length of 31,371,815 bp. Benchmarking Universal Single Copy Orthologs analysis revealed 98.0% complete orthologs, indicative of a high-quality assembly representative of genic space. Annotation of protein-coding sequences revealed 58,702 high-confidence genes encoding 88,528 gene models. Access to the ‘Munstead’ genome will permit comparative analyses within and among lavender accessions and provides a pivotal species for comparative analyses within Lamiaceae.

## Objective

Lavender (*Lavandula*) of Lamiaceae, or the mint family, comprises nearly 50 species, a number of which are used as ornamentals, culinary herbs, and/or sources of essential oils. Lavender produces a rich array of chemical compounds that largely serve a defensive role in nature to deter herbivory. Lavender oil, for example, contains roughly 100 different chemical compounds; a large part of this secondary metabolite diversity is primarily due to the diversity of terpenes (e.g., monoterpenes, sesquiterpenes, diterpenes). The most widely grown and commercially important species is *Lavandula angustifolia* (English lavender), grown as both an ornamental and a source of oil, but *Lavandula* contains a wide range of cultivated species and hybrids; for example, *L. stoechas*, *L. dentata*, and *L. multifida* are widely grown as ornamentals. In addition, a hybrid between *L. angustifolia* and *L. latifolia* (*Lavandula* × *intermedia*), Dutch lavender, is grown commercially. Essential oils of *L. angustifolia* are widely used in various medicines, including balms and salves, as well as in perfumes and cosmetics. Although lavender is of great economic and ornamental importance, its underlying chemical diversity remains poorly understood. To help elucidate the genetic control of the rich chemical diversity of lavender, we report here the sequencing and chromosome-scale assembly and annotation of the complete nuclear genome of *L. angustifolia* cv. ‘Munstead’. Two other genome assemblies of *L. angustifolia* have been reported, from the ‘Maillette’ [1] and ‘Jingxun 2’ [2] cultivars as well as from *L.* × *intermedia* ‘Super’ [3]. Given the large number of lavender cultivars, access to the ‘Munstead’ genome will be of further utility by facilitating comparative analyses of the genetic basis of chemodiversity within this commercially important species.

## Data description

*Lavandula angustifolia* ‘Munstead’ was obtained from Van Atta’s nursery (Bath, MI, USA) and grown in a growth chamber under 300 μE light intensity, 70% relative humidity, 15 h day length, 23.3 °C day, and 13.8 °C night. Flow cytometry of Munstead leaf tissue revealed an estimated genome size (1C) of 907 Mb, and a chromosome squash of root tips [4] revealed 50 chromosomes, indicating a haploid chromosome number of 25 (Table 1, Data file 1, [5]). High-molecular-weight DNA was isolated from immature leaves of a single ‘Munstead’ plant using a modified CTAB isolation protocol followed by a Qiagen Genomic Tip and an Amicon buffer exchange as described previously [6]. High-molecular-weight DNA was used to construct libraries for sequencing using the Oxford Nanopore Technologies (ONT) and Illumina platforms (Table 1, Data file 2, Data sets 1–16, [5, 7–22]). For whole genome shotgun sequence data, a single Illumina TruSeq Nano DNA library was constructed and sequenced on a HiSeq 4000 in paired-end mode, generating ~ 310 M paired-end 150-nt reads (Table 1, Data file 2, Data set 16, [5, 22]). These reads were used to estimate heterozygosity using Genomescope (v2.0) [23] using the diploid model in which the observed k-mer distribution did not match the expected model (Table 1, Data file 3, [5]). Estimation of ploidy using Smudgeplot [23] suggested Munstead was a tetraploid genome as shown by the allele distribution (Table 1, Data file 3, [5]). Next, a single SQK-LSK108 and 14 SQK-LSK109 ONT Ligation Sequencing libraries were constructed and sequenced on five FLO-MIN106 and 10 FLO-MIN106 Rev D ONT flow cells (Table 1, Data file 2, Data sets 1–15, [5, 7–21]). ONT genomic DNA reads were base called using Guppy (v3.4.1) and reads less than 10 kb removed. The final dataset used for assembly included 2,953,837 reads (80.4 Gb), providing an estimated 89 × coverage of the genome. Reads were assembled using Canu (v1.9) [24] with the options: minOverlapLength = 4000 and genomeSize = 980 m. The contigs were error corrected using two rounds of Racon (v1.4.10) [25] followed by two rounds of Medaka (v0.11.5) [26] and two rounds of Pilon (v1.23) [27]. Due to the heterozygosity of the Munstead genome, haplotigs were removed through two rounds of purge_dups (v1.0.0) [28]. Hi-C Illumina HiSeq 4000 paired-end 150-nt reads were generated from two Dovetail Hi-C [29] libraries constructed from immature leaves (Table 1, Data files 2 & 4, Data sets 17 & 18, [5, 30, 31]) and used to scaffold the contigs into 25 chromosomes using Juicer (v1.6) [32] and 3D-DNA (git commit: 529ccf4) [33]. To screen the final contigs for contamination, contigs were split into 10-kb windows and searched against the NCBI nt database using Centrifuge (v1.0.4-beta; [34]); no full contigs or regions were identified as contaminants. The final assembly is 795,075,733 bp with an N50 scaffold length of 31,371,815 bp (Table 1, Data files 5 & 6, Data set 19, [5, 35]). Estimation of k-mer representation in the assembly using KAT (v3.4.1) [36] revealed nearly complete purging of haplotigs (Table 1, Data file 7, [5]) although k-mers not represented in the assembly suggest that lavender is a diploidized autopolyploid in which a subset of the alleles are heterozygous. Benchmarking Universal Single Copy Orthologs (BUSCO, v5.4.3) [37] analysis of the genome assembly revealed 98.0% complete BUSCOs and 68.3% complete and duplicated BUSCOs (Table 1, Data file 8, [5]) indicating a near-complete genic space consistent with a polyploid ancestry and recent diploidization of lavender.

**Table 1:** Overview of data files and data sets used in this study

Label	Data file/Data set name	File types	Data repository and identifier (DOI or accession number), Citation Number
Data file 1	Chromosome squash of *Lavendula angustifolia* root tips	Portable Network Graphic (.png)	Figshare (https://doi.org/10.6084/m9.figshare.23982972.v3) [5]
Data file 2	*Lavendula angustifolia* libraries used in this study	Spreadsheet (.xlxs)	Figshare (https://doi.org/10.6084/m9.figshare.23982972.v3) [5]
Data file 3	k-mer frequency distribution plot and ploidy estimation	Portable Document Files (.pdf)	Figshare (https://doi.org/10.6084/m9.figshare.23982972.v3) [5]
Data file 4	Hi-C contact map	Portable Document Files (.pdf)	Figshare (https://doi.org/10.6084/m9.figshare.23982972.v3) [5]
Data file 5	Assembly metrics for the *Lavandula angustifolia* assembly	Spreadsheet (.xlxs)	Figshare (https://doi.org/10.6084/m9.figshare.23982972.v3) [5]
Data file 6	Pseudomolecule lengths and gap content for the *Lavandula angustifolia* assembly	Spreadsheet (.xlxs)	Figshare (https://doi.org/10.6084/m9.figshare.23982972.v3) [5]
Data file 7	k-mer comparison plot	Portable Document Files (.pdf)	Figshare (https://doi.org/10.6084/m9.figshare.23982972.v3) [5]
Data file 8	BUSCO results on the *Lavandula angustifolia* assembly and annotation	Spreadsheet (.xlxs)	Figshare (https://doi.org/10.6084/m9.figshare.23982972.v3) [5]
Data file 9	Repetitive sequence content in the *Lavandula angustifolia* assembly	Spreadsheet (.xlxs)	Figshare (https://doi.org/10.6084/m9.figshare.23982972.v3) [5]
Data file 10	*Lavandula angustifolia* gene annotation summary	Spreadsheet (.xlxs)	Figshare (https://doi.org/10.6084/m9.figshare.23982972.v3) [5]
Data set 1	Oxford Nanopore Technologies High molecular weight genomic DNA, SRR15929008	Fastq file (.fastq.gz)	NCBI (https://identifiers.org/ncbi/insdc.sra:SRR15929008) [7]
Data set 2	Oxford Nanopore Technologies High molecular weight genomic DNA, SRR15929007	Fastq file (.fastq.gz)	NCBI (https://identifiers.org/ncbi/insdc.sra:SRR15929007) [8]
Data set 3	Oxford Nanopore Technologies High molecular weight genomic DNA, SRR15929001	Fastq file (.fastq.gz)	NCBI (https://identifiers.org/ncbi/insdc.sra:SRR15929001) [9]
Data set 4	Oxford Nanopore Technologies High molecular weight genomic DNA, SRR15929000	Fastq file (.fastq.gz)	NCBI (https://identifiers.org/ncbi/insdc.sra:SRR15929000) [10]
Data set 5	Oxford Nanopore Technologies High molecular weight genomic DNA, SRR15928999	Fastq file (.fastq.gz)	NCBI (https://identifiers.org/ncbi/insdc.sra:SRR15928999) [11]
Data set 6	Oxford Nanopore Technologies High molecular weight genomic DNA, SRR15928998	Fastq file (.fastq.gz)	NCBI (https://identifiers.org/ncbi/insdc.sra:SRR15928998) [12]
Data set 7	Oxford Nanopore Technologies High molecular weight genomic DNA, SRR15928997	Fastq file (.fastq.gz)	NCBI (https://identifiers.org/ncbi/insdc.sra:SRR15928997) [13]
Data set 8	Oxford Nanopore Technologies High molecular weight genomic DNA, SRR15928996	Fastq file (.fastq.gz)	NCBI (https://identifiers.org/ncbi/insdc.sra:SRR15928996) [14]
Data set 9	Oxford Nanopore Technologies High molecular weight genomic DNA, SRR15928995	Fastq file (.fastq.gz)	NCBI (https://identifiers.org/ncbi/insdc.sra:SRR15928995) [15]
Data set 10	Oxford Nanopore Technologies High molecular weight genomic DNA, SRR15928994	Fastq file (.fastq.gz)	NCBI (https://identifiers.org/ncbi/insdc.sra:SRR15928994) [16]
Data set 11	Oxford Nanopore Technologies High molecular weight genomic DNA, SRR15929006	Fastq file (.fastq.gz)	NCBI (https://identifiers.org/ncbi/insdc.sra:SRR15929006) [17]
Data set 12	Oxford Nanopore Technologies High molecular weight genomic DNA, SRR15929005	Fastq file (.fastq.gz)	NCBI (https://identifiers.org/ncbi/insdc.sra:SRR15929005) [18]
Data set 13	Oxford Nanopore Technologies High molecular weight genomic DNA, SRR15929004	Fastq file (.fastq.gz)	NCBI (https://identifiers.org/ncbi/insdc.sra:SRR15929004) [19]
Data set 14	Oxford Nanopore Technologies High molecular weight genomic DNA, SRR15929003	Fastq file (.fastq.gz)	NCBI (https://identifiers.org/ncbi/insdc.sra:SRR15929003) [20]
Data set 15	Oxford Nanopore Technologies High molecular weight genomic DNA, SRR15929002	Fastq file (.fastq.gz)	NCBI (https://identifiers.org/ncbi/insdc.sra:SRR15929002) [21]
Data set 16	Illumina WGS DNA, SRR15915200	Fastq file (.fastq.gz)	NCBI (https://identifiers.org/ncbi/insdc.sra:SRR15915200) [22]
Data set 17	Illumina Hi-C DNA, SRR15931069	Fastq file (.fastq.gz)	NCBI (https://identifiers.org/ncbi/insdc.sra:SRR15931069) [30]
Data set 18	Illumina Hi-C DNA, SRR15931068	Fastq file (.fastq.gz)	NCBI (https://identifiers.org/ncbi/insdc.sra:SRR15931068) [31]
Data set 19	Genome assembly of *Lavandula angustifolia*	fasta file (.fa)	NCBI (https://identifiers.org/assembly/GCA_028984105) [35]
Data set 20	Illumina RNA-seq: RNA-seq-mature leaf, SRR15915199	Fastq file (.fastq.gz)	NCBI (https://identifiers.org/ncbi/insdc.sra:SRR15915199) [38]
Data set 21	Illumina RNA-seq: immature leaf, SRR15915191	Fastq file (.fastq.gz)	NCBI (https://identifiers.org/ncbi/insdc.sra:SRR15915191) [39]
Data set 22	Illumina RNA-seq: inflorescence, SRR15915190	Fastq file (.fastq.gz)	NCBI (https://identifiers.org/ncbi/insdc.sra:SRR15915190) [40]
Data set 23	Illumina RNA-seq: stem, SRR15915189	Fastq file (.fastq.gz)	NCBI (https://identifiers.org/ncbi/insdc.sra:SRR15915189) [41]
Data set 24	High Confidence Gene Models cDNA	fasta file (.fa)	Figshare (https://doi.org/10.6084/m9.figshare.23982972.v3) [5]
Data set 25	High Confidence Gene Models CDS	fasta file (.fa)	Figshare (https://doi.org/10.6084/m9.figshare.23982972.v3) [5]
Data set 26	High Confidence Gene Models GFF3	GFF3 file (.gff3)	Figshare (https://doi.org/10.6084/m9.figshare.23982972.v3) [5]
Data set 27	High Confidence Gene Models Proteins	fasta file (.fa)	Figshare (https://doi.org/10.6084/m9.figshare.23982972.v3) [5]
Data set 28	Representative High Confidence Gene Models cDNA	fasta file (.fa)	Figshare (https://doi.org/10.6084/m9.figshare.23982972.v3) [5]
Data set 29	Representative High Confidence Gene Models CDS	fasta file (.fa)	Figshare (https://doi.org/10.6084/m9.figshare.23982972.v3) [5]
Data set 30	Representative High Confidence Gene Models GFF3	GFF3 file (.gff3)	Figshare (https://doi.org/10.6084/m9.figshare.23982972.v3) [5]
Data set 31	Representative High Confidence Gene Models List	text file (.txt)	Figshare (https://doi.org/10.6084/m9.figshare.23982972.v3) [5]
Data set 32	Representative High Confidence Gene Models Proteins	fasta file (.fa)	Figshare (https://doi.org/10.6084/m9.figshare.23982972.v3) [5]
Data set 33	Working Gene Models cDNA	fasta file (.fa)	Figshare (https://doi.org/10.6084/m9.figshare.23982972.v3) [5]
Data set 34	Working Gene Models CDS	fasta file (.fa)	Figshare (https://doi.org/10.6084/m9.figshare.23982972.v3) [5]
Data set 35	Working Gene Models GFF3	GFF3 file (.gff3)	Figshare (https://doi.org/10.6084/m9.figshare.23982972.v3) [5]
Data set 36	Working Gene Models Proteins	fasta file (.fa)	Figshare (https://doi.org/10.6084/m9.figshare.23982972.v3) [5]
Data set 37	Working Gene Models Functional Annotation	text file (.txt)	Figshare (https://doi.org/10.6084/m9.figshare.23982972.v3) [5]
Data set 38	*Lavandula angustifolia* genome sequence	fasta file (.fa)	Figshare (https://doi.org/10.6084/m9.figshare.23982972.v3) [5]

Data files describing the assembly, annotation, and analysis of the Munstead genome are provided. Datasets include raw genome and transcriptome datasets, genome assembly, and annotation files

A custom repeat library was created using RepeatModeler (v2.0.1) [42, 43] and used to mask the genome (65.2% masked, Table 1, Data file 9, [5]) prior to annotation with RepeatMasker (v4.1.0) [44]. To provide empirical transcript evidence for annotation, RNA from three plants was isolated from a tissue panel (immature leaf, mature leaf, inflorescence, and stem) using the hot phenol extraction method as described previously [45]. Illumina TruSeq Stranded mRNA libraries were constructed and sequenced on an Illumina HiSeq 4000, generating 150-nt paired-end reads (Table 1, Data file 2, Data sets 20–24, [5, 38–41]). Reads were cleaned of adaptors and low-quality sequences using Cutadapt (v2.9) [46] and then aligned to the genome assembly with HISAT2 (v2.2.0) [47] and assembled using Stringtie (v2.1.1) [48] to generate genome-guided transcript assemblies. Ab initio gene models were created using the BRAKER2 (v2.1.5) [49] pipeline using the RNA-seq alignments as hints and then refined using PASA (v2.4.1) [50, 51]. Functional annotation was assigned to the working gene models using matches to the *Arabidopsis* predicted proteome (TAIR10) [52], Pfam domains [53], and SwissProt plant protein sequences as described previously [43]. High-confidence models within the working model set were defined based on the presence of a Pfam domain and/or gene expression data as defined as a transcript per million greater than zero. Representative gene models were defined as the gene model with the longest coding sequence at a locus. The final working gene set is composed of 68,432 genes and 98,924 gene models (Table 1, Data file 10, [5]). The high-confidence set is composed of 58,702 genes and 88,528 gene models (Table 1, Data file 10, [5]). BUSCO analyses of the gene models revealed > 95% complete BUSCOs (Table 1, Data file 8, [5]) in the working, high-confidence, and high-confidence representative gene model sets including high-quality annotation of the ‘Munstead’ genome.

## Limitations

The estimated genome size of ‘Munstead’ is greater than the final, error-corrected assembly, suggesting that we are missing portions of the genome. Due to sequence complexity, it is likely that highly repetitive sequences within the centromeric and pericentromeric regions are under-represented in the assembly. K-mer estimations of heterozygosity suggest the ‘Munstead’ genome is heterozygous, which is consistent with the presence of haplotigs in the initial Canu assembly. By purging duplicates to generate a haploid assembly, we may have removed structural variants such as diverged paralogs or presence/absence variants from the final assembly. To prevent over-annotation of pseudogenes, our annotation pipeline is dependent on empirical support and/or similarity to annotation proteins and Pfam domains. Thus, it is possible that genes shorter than our minimum length (50 amino acids) and/or that lack expression or protein evidence are not present in our annotated gene sets.

## Data Availability

The data described in this Data note can be freely and openly accessed at the National Center for Biotechnology Information Short Read Archive under BioProject ID PRJNA762277 (https://identifiers.org/bioproject:PRJNA762277; [35]). The assembled genome is available in GenBank under the accession JAPVEC000000000 (https://identifiers.org/assembly:GCA_028984105.1; [35]) and on Figshare [5]. A summary of data sets available on Figshare [5] is available in Table 1. A voucher specimen is available at the Michigan State University Herbarium (MSC0273865).

## Abbreviations

BUSCO: Benchmarking Universal Single Copy Orthologs
ONT: Oxford Nanopore Technologies
PASA: Program to Assemble Spliced Alignments
